# Parliament and the Professions

**Published:** 1921-07-30

**Authors:** 


### Parliament and the Professions.

### Anthrax-Infected Brushes.

The Minister of Health informed Major Kelley that samples of imported brushes from various sources have been examined from time to time, but so far only shaving-brushes of Japanese origin have been found to be anthrax-infected and capable of conveying infection to human beings. Importation of these brushes has been prohibited since February 1920, and precautions have been taken to see that they do not come through other countries.

### Death Certificates.

Mr. Walterson asked the Minister of Health how many deaths were certified by registered practitioners during each year for the past ten years; how many deaths were uncertified; how many of the latter deaths were reported to coroners. Sir Alfred Mond replied that it is not possible to state the number of deaths certified or uncertified. The available statistics are arrived at from deaths registered; deaths which are the subject of an inquest are registered on the certificate of the coroner, whether they had or had not been previously certified by a registered practitioner. The figures of deaths registered respectively in each of these groups were submitted by the Minister in a table giving detailed information for ten years. In the year 1920 429,426 deaths were registered on a certificate of a registered practitioner, 30,995 were registered on the coroner's certificate after an inquest, and 5,709 were uncertified on which no inquest was held.

### Awards for Medical Research.

Mr. Balfour answered a question addressed to the Prime Minister by Mr. Briant with regard to the possibility of a fund from which awards could be paid for discoveries or inventions which contribute to the general health of the community, and which are placed gratuitously at the service of the public. He said he could see no grounds for differentiating medical from other forms of research benefiting the public, and he doubted if any pecuniary assistance in such awards would be beneficial to science or medicine. The question had been discussed at a deputation he had received last year. With regard to persons who had lost their lives through research into the value of x-rays, their dependants were eligible for help from the Royal Bounty Fund, but whether that fund is large enough is a question upon which he did not feel able to give an opinion.

